# Ethical Aspects concerning Instrument Separation and Perforations during Endodontic Treatment: A Cross-Sectional Study

**DOI:** 10.1155/2020/8849105

**Published:** 2020-09-15

**Authors:** Raidan Ba-Hattab, Ishrat Rahman, Lubna K. Elsayed, Wejdan F. Alasmari, Randa Abidia, Somayah Abdelgaffar, Awsan Bahattab

**Affiliations:** ^1^College of Dental Medicine, Qatar University, Doha, Qatar; ^2^Department of Basic Dental Sciences, College of Dentistry, Princess Nourah Bint Abdulrahman University, Riyadh, Saudi Arabia; ^3^Department of Oral Surgery, Faculty of Dentistry, Suez Canal University, Ismaielia, Egypt; ^4^Private Sector, Riyadh, Saudi Arabia; ^5^Department of Preventive Dental Sciences, Dar Al Uloom University, Riyadh, Saudi Arabia; ^6^Private Sector, Jeddah, Saudi Arabia; ^7^CRIMEDIM–Research Center in Emergency and Disaster Medicine, Università Del Piemonte Orientale, Novara, Italy

## Abstract

**Aim:**

During endodontic treatment, dentists may face various unwanted procedural accidents, at any stage of the treatment that might compromise endodontic treatment outcome and bring obstacles to dentists as well. This study aimed to address and analyze several ethical concerns relating to the behavioural conduct of dentists towards endodontic instrument separation as well as perforation of the crown and/or root during root canal treatment in Riyadh, Saudi Arabia.

**Method:**

Hundred and eleven questionnaires were distributed among dentists working in Riyadh in university clinics and government and private sectors. Data were collected, reviewed, and statistically analyzed by Fisher's exact and chi-square tests at a 5% significance level, using SPSS software.

**Results:**

54.5% of the respondents have encountered instrument separation. 53.2% stated that they would inform the patient about the instrument separation. 43.6% of the respondents had experienced perforation during root canal treatment, and 54.9% reported that they would inform the patient of the accident.

**Conclusion:**

Within the limitation of this survey, we concluded that most of the dental professionals did not hesitate to adhere to the correct ethical conduct, and they would inform the patient if an incident occurred.

## 1. Introduction

Dental malpractice is a careless, unintended act of dental professional who fails to follow the standards of care resulting in harming the patient [1]. Approximately 14%–17% [2, 3] of all dental malpractices comprise endodontic malpractices. Despite having a growing number of latest techniques and novel inventions to improve the efficiency of endodontic treatment, in recent years, an increasing number of negligence cases have also been reported [4]. The most common malpractices in endodontic are perforation and a broken instrument [5].

In order to establish a sound and safe patient relationship, it is essential to adhere to basic legal and ethical dental practices and principles where dental ethics must be the guideline in cases of an accident such as instrument separation. Proper explanations before treatment (i.e., appropriate informed consent) might save grief both to the patient and to the dentist [6–8]. However, when an accident does occur, the dentist must inform the patient, so the patient is aware of the possible consequences, treatment options, and prognosis. It is also advisable to provide complete documentation of the case to the patient [9]. To reduce the accidents rates in endodontic treatment, dentists should strictly adhere to the standards of healthcare [10] and adopt the prevention strategies [11–13].

A well-acknowledged statement by Cohen [14] mentions that care and skill in a dental practitioner do not guarantee the absence of mishaps and fracture of the endodontic instrument during root canal treatment.

Only one study has investigated ethical considerations and endodontic malpractices in Saudi Arabia [15], and since there has recently been an increasing number of dental graduates in Saudi Arabia, we aimed in this study to address the ethical aspects concerning the behaviour of dentists regarding endodontic instrument separation as well as perforation of the crown and/or root during root canal treatment in Riyadh. The null hypothesis was that there was no association between the specialty of the dentist and their conduct in response to instrumental separation and perforations during endodontic treatment.

## 2. Materials and Methods

The research was revised and approved by the research committee at the college of dentistry, Princess Nourah Bint Abdulrahman University, Riyadh. The questionnaire was designed and structured, containing ten close-ended questions modified and framed, based on da Silva et al. [4]. Four questions were related to the participant's demographic data like age, gender, specialty, and place of work, and six questions were related to the admission of the mishap as well as the conduct of the dentists when the malpractice was perceived (Table 1). A total of 111 questionnaires were distributed among dentists working in Riyadh (general dentists, endodontists, and other dentists with different specialties) in governmental, private, and university clinics. The sample size was calculated assuming that the expected behavioural misconduct of the dentist was 50% using Lwanga and Lemeshow practical manual [16] with absolute precision of 10% and 95% confidence interval. A further 15% of the calculated sample size was added to compensate for nonresponse rate; thus, the total sample size was 111. The questionnaire was disturbed as a hard-printed copy.

### 2.1. Statistical Analysis

Data were entered and analyzed by using SPSS (version 20.0) software. Categorical variables were described using frequency distribution and percentage. Comparison of variables was performed using the chi-square test or Fisher's exact test if necessary. *p* value < 0.05 was considered statistically significant.

## 3. Results

Of the 111 questionnaires distributed, 110 were obtained, which is a response rate of 99.1%. Based on data collected from the replied questionnaires, 60% (*n* = 66) of the respondents were general dentists, 9.1% (*n* = 10) were endodontists, and 30.9% (*n* = 34) were other dental specialists (Table 2).

### 3.1. Experience of Instrument Separation and Perforation

The result in Table 3 shows that 54.5% (*n* = 60) of the respondents had experienced instrument separation during root canal treatment, and there was a statistically significant correlation seen between professional qualification and fracture occurrence (*p* ≤ 0.002). However, 43.6% (*n* = 48) of the respondents had experienced perforation during root canal treatment, and there was no statistically significant correlation between professional qualification and perforation occurrence (*p* ≥ 0.05).

### 3.2. Instrument Separation with the Possibility of Removal

The result in Table 4 displays the respondents' conduct if instrument separation occurs during root canal treatment with possibilities of fragment removal. When questioned about their initial conduct in the case of instrument separation with the possibilities of instrument removal, 53.2% (*n* = 33) of respondents stated that they would inform the patient about the accident, 22.6% (*n* = 14) would inform the patient and refer to another professional, 12.9% (*n* = 8) would inform the patient with no attempt to remove the fragment, and 4.8% (*n* = 3) would not inform the patient and continue the treatment. Only 1.6% (*n* = 1) would not inform the patient and would refer them to another professional. 4.8% (*n* = 3) of the respondents would have another conduct. The difference among the groups was not statistically significant (*p* ≥ 0.05).

### 3.3. Instrument Separation with No Possibility of Removal

The results in Table 5 display the respondents conduct if instrument separation occurs during root canal treatment with no possibilities of fragment removal. When questioned about their initial conduct in case of instrument separation with no possibilities of instrument removal, 19% (*n* = 12) stated that they would inform the patient about the accident and try to remove the fragment in the next visit, and 6.6% (*n* = 4) would not inform the patient and continue the dental treatment. Only 1.6 (*n* = 1) would not inform the patient and would refer them to an endodontist. 1.6% (*n* = 1) of the respondents would have another conduct. There was no statistically significant difference among the groups concerning their conduct towards the patient (*p* ≥ 0.25).

### 3.4. Perforation with Good Prognosis

The results in Table 6 display the respondents' conduct if perforation occurs during root canal treatment with good prognosis. When the participants were asked about their initial conduct in case of perforation with a good prognosis, 54.9% (*n* = 28) stated that they would inform the patient about the accident and would try to repair it in the same appointment. 17.6% (*n* = 9) would inform the patient and refer to another professional. 9.8% (*n* = 9) would continue the treatment without informing the patient, 9.8% (*n* = 5) would not inform the patient and neither refer them to endodontists, and 5.9% (*n* = 3) would inform the patient and postpone the repair to another visit. 2% (*n* = 1) of the respondents would have another conduct. There was no statistically significant difference among the groups concerning their conduct towards the patient concerning perforation with good prognosis (*p* ≥ 0.597).

### 3.5. Perforation with Poor Prognosis

The results in Table 7 display the respondents' conduct if perforation occurs during root canal treatment with poor prognosis. When the participants were asked about their initial conduct in the case of perforation with poor prognosis, 40% (*n* = 33) stated that they would inform the patient about the accident and would refer to another professional. 32% (*n* = 16) of the participants would inform the patient and try to repair it in the same appointment. 8% (*n* = 4) would inform the patient and postpone the repair to another visit, and 8% (*n* = 6) would inform the patient and refer to another professional. However, 6% (*n* = 9) would continue the treatment without telling the patient. 6% of the participants (*n* = 9) would have another conduct. No statistically significant difference was observed among the groups concerning their conduct towards the patient concerning perforation with poor prognosis (*p* ≥ 0.229).

## 4. Discussion

During routine endodontic therapy, clinicians can experience situations, at any stage of treatment, where accidents occur, and obstacles have to be overcome [9]. However, hiding the procedural accident from the patient is considered as negligence that exposes the dentist to litigations [17]. Careless endodontic diagnosis or treatment could be avoided by adhering to the standard care.

A negative effect on the long-term prognosis is foreseeable in the case of intracanal instrument separation [18]. In the present study, more than half of the participants (54.5%) had encountered instrument separation during root canal treatment, and this proportion is far less than which was previously reported in Riyadh (88%) [15]. The disparity may reflect an overall increase in proficiency of the dentists in Riyadh over the last years or may be an artefact of a smaller sample size. A previous study in Riyadh showed that 93% of all endodontists had experienced instrument separation [15]. The current study is in full agreement to this where all of the endodontists 9.1% (*n* = 10) of the total respondents had experienced the occurrence of instrument separation. These data show that even a specialist, who is presumably more skilled and technically prepared than a general dentist, might experience this type of accident. A separated endodontic file in the canal may cause anxiety, anguish, and agony to the patient. Such experience and patient's dissatisfaction may change patient's attitude toward dental treatment causing dental fear [19], which is a common problem in children and adolescents among several countries in Europe, Asia, Africa, and North America [20].

It is an embarrassing situation for dentists to face the patient once such a mishap occurs unless the dentist had explained to the patient about the complexity of the root canal treatment and its potential complications prior to initiation of treatment [21, 22].

The clinicians have a legal obligation to inform the patient and to document it in the patient's notes—if an instrument has fractured during treatment [22]. However, only approximately half of the participants (53.2%) had identified that they would inform the patient about a fractured instrument if there was a possibility of removal. Thus, there is a significant degree of hesitation in informing patients about the mishap, as previously reported [4, 15].

Possible options, in the management of a case of instrument separation in the root canal, are leave, bypass, or remove [23]. A thorough assessment of the likely prognosis in all cases, based on the potential benefit of removal and the likelihood and risk of complication, is the sole factor by which clinicians should take their management decision [22]. In the present study, most endodontists (70%) had confirmed that they would inform the patient about the accident and would try to remove the instrument in another visit, and this correlates well to previously reported value (79.3%) [15]. Several factors need to be considered in the management of separated files. For instance, the factors to consider in the removal of a fractured NiTi instrument are strategic importance of the tooth, any periapical disease the clinician experiences, other tooth and patient factors, as well as the availability and use of equipment, instruments, and techniques [24]. Chances of improved removal have significantly increased with the application and incorporation of microscopes, fine ultrasonic tips, and staging platforms [25, 26]. Furthermore, there is an improvement in the management of the separated files with the increasing years of clinical experience [27].

In the case of instrument separation with no possibilities of removal, approximately half of the general practitioners (48.4%) would inform the patients and refer them to an endodontist; this is a greater proportion in Riyadh than previously reported in Brazil (34.8%) [4]. Our data identify that approximately half of all general practitioners in Riyadh are aware of their limitation regarding their technical capacity; it also indicates their concern to provide the best care possible to the patient since an endodontist would handle the case. On the contrary, half of the endodontists would inform the patient and continue the treatment with no attempt to remove the fragment. In such a case, in order to prevent the development of any associated periapical pathology, periodic radiographic review would be necessary [22] as the best option in some cases of instrument separation may be to leave the fractured instrument [19–22, 25–30] prescribed with regular follow-up. One particular limitation of this questionnaire-based research is the lack of case-based questions. Assessing the responses of endodontists in various clinical scenarios would highlight the proficiency of the endodontists in recognizing real cases. In addition, it should be identified whether leaving the instrument fragment would be the best management option for the patient.

Regarding perforation, our study identifies that less than half (43.6%) of participants had encountered perforation during root canal treatment. Perforation can lead to an increased risk of oral infection. Factors that affect the risk of infection at perforation sites are size and shape of the perforation, the location of perforation, and the time taken till detection [31]. Correspondingly, a small perforation should be detected promptly and managed rapidly at the time of the occurrence by sealing with intracanal sealants (MTA or other bioceramic repair materials) placed at the correct location under good visibility, and necessary magnification increases the management success rate [32, 33].

The present study showed that the majority of endodontists will resolve the problem immediately in case of good prognosis and will inform the patients, which reflects their expertise and awareness.

Bacterial infection around the surrounding area of perforation is most likely inevitable if a delay beyond 24–72 hours occurs in diagnosis and or treatment [33]. In the complication of a delay in management, and a subsequent infection arises, immediate extraction of the infected tooth may become necessary [31]. The present study showed that half of the general dentists would refer the patient to a specialist in case of poor prognosis, as they know their limitations. The American Association of Endodontists has provided an assessment form to evaluate the case difficulty. This form is used to envisage the case complexity and mishaps that the dentists could encounter during root canal treatment [34]. All dental practitioners need to be judicious in referring cases to an endodontic specialist by recognizing the limits of their skill, expertise, competency, and experience [10, 35]. To provide optimum patient safety and care and to avoid substandard patient care, a program of continuing professional education is required, as dental procedures are technically sensitive that need extensive training and knowledge as well as cognitive and psychomotor skills [10, 36].

## 5. Conclusion

The majority of dentists in Riyadh had experienced endodontic accidents during root canal treatment. Most of them did not hesitate to do the correct ethical conduct, which is important to maintain the confidence between the patient and dentist and avoid future litigations.

## Figures and Tables

**Table 1:** Questions related to the admission of the mishap as well as the conduct of the dentists when the malpractice was perceived.

1. *Have you ever fractured any type of endodontic instrument?*
(a) Yes
(b) No
(c) Not sure
2. *What is your conduct when intracanal breakage of an instrument occurs with possibilities of fragment removal?*
(a) Inform the patient and finish the treatment in another appointment (try to remove the fragment)
(b) Inform the patient and continue the treatment (no attempt to remove the fragment)
(c) Inform the patient and refer to another professional
(d) Do not inform the patient and continue the treatment
(e) Do not inform the patient and refer to another professional
(f) Other conduct, state
3. *What is your conduct when intracanal breakage of an instrument occurs with no possibilities of fragment removal?*
(a) Inform the patient and finish the treatment in another appointment (try to remove the fragment)
(b) Inform the patient and continue the treatment (no attempt to remove the fragment)
(c) Inform the patient and refer to another professional
(d) Do not inform the patient and continue the treatment
(e) Do not inform the patient and refer to another professional
(f) Other conduct, state
4. *Have you ever perforated a canal and/or a crown during root canal treatment?*
(a) Yes
(b) No
(c) Not sure
5. *What is your conduct when you perforate a canal and/or a crown (perforation with a good prognosis) while you prepare it for root canal treatment?*
(a) Inform the patient and repair it immediately
(b) Inform the patient and repair it in another appointment
(c) Inform the patient and refer to another professional
(d) Do not inform the patient and continue the treatment
(e) Do not inform the patient and refer to another professional
(f) Other conduct, state
6. *What is your conduct when you perforate a canal and/or a crown (perforation with a poor prognosis) while you prepare it for root canal treatment?*
(a) Inform the patient and repair it immediately
(b) Inform the patient and repair it in another appointment
(c) Inform the patient and refer to another professional
(d) Do not inform the patient and continue the treatment
(e) Do not inform the patient and refer to another professional
(f) Other conduct, state

**Table 2:** Frequency of dental specializations.

Variables	Frequency	Percent (%)
General dentist	66	60
Endodontist	10	9.1
Other dental specialties	34	30.9
Total	110	100

**Table 3:** Percentage of respondents who had experienced instrument separation and/or perforation during previous root canal treatment.

Area of specialization	Instrument separation	Perforation
General dentists	25.5% (*n* = 28)	23.6% (*N* = 26)
Endodontists	9.1% (*n* = 10)	6.4% (*N* = 7)
Other dental specialists	20.0% (*n* = 22)	13.6% (*N* = 15)
Total	54.5% (*n* = 60)	43.6% (*N* = 48)
*p* value	0.002	0.493

**Table 4:** Frequency of respondents when related to their conduct in case of instrument separation with the possibility of fragment removal (*p* ≥ 0.05).

Instrument separation with the possibility of removal	General dentist	Endodontists	Other dental specialists
Inform the patient and finish the treatment in another appointment (try to remove the fragment)	40% (*n* = 12)	70% (*n* = 7)	63.6% (*n* = 14)
Inform the patient and continue the treatment (no attempt to remove the fragment)	13.3% (*n* = 4)	20% (*n* = 2)	9.1% (*n* = 2)
Inform the patient and refer to another professional	33.3% (*n* = 10)	0.0% (*n* = 0)	18.2% (*n* = 4)
Do not inform the patient and continue the treatment	6.7% (*n* = 2)	10% (*n* = 1)	0.0% (*n* = 0)
Do not inform the patient and refer to another professional	3.3% (*n* = 1)	0.0% (*n* = 0)	0.0% (*n* = 0)
Other conduct	3.3% (*n* = 1)	0.0% (*n* = 0)	9.1% (*n* = 2)

**Table 5:** Frequency of respondents when related to their conduct in case of instrument separation with the possibility of fragment removal (*p* ≥ 0.25).

Instrument separation with no possibility of removal	General dentist	Endodontists	Other dental specialists
Inform the patient and finish the treatment in another appointment (try to remove the fragment)	16.1% (*n* = 5)	40% (*n* = 4)	15% (*n* = 3)
Inform the patient and continue the treatment (no attempt to remove the fragment)	19.4% (*n* = 6)	50% (*n* = 5)	50% (*n* = 10)
Inform the patient and refer to another professional	48.4% (*n* = 15)	0.0%	35% (*n* = 7)
Do not inform the patient and continue the treatment	9.7% (*n* = 3)	10% (*n* = 1)	0.0%
Do not inform the patient and refer to another professional	3.2% (*n* = 1)	0.0%	0.0%
Other conduct	3.2% (*n* = 1)	0.0%	0.0%

**Table 6:** Frequency of respondents when related to their conduct in case of perforation with good prognosis (*p* ≥ 0.597).

Perforation with good prognosis	General dentist	Endodontists	Other dental specialists
Inform the patient and repair it immediately	53.6% (*n* = 15)	83.3% (*n* = 5)	47.1% (*n* = 8)
Inform the patient and repair it in another appointment	7.1% (*n* = 2)	0%	5.9% (*n* = 1)
Inform the patient and refer to another professional	21.4% (*n* = 6)	0.0%	17.6% (*n* = 3)
Do not inform the patient and continue the treatment	3.6% (*n* = 1)	16.7% (*n* = 1)	17.6% (*n* = 3)
Do not inform the patient and refer to another professional	14.3% (*n* = 4)	0.0%	5.9% (*n* = 1)
Other conduct	0.0%	0.0%	5.9% (*n* = 1)

**Table 7:** Frequency of respondents when related to their conduct in case of perforation with poor prognosis (*p* ≥ 0.229).

Perforation with poor prognosis	General dentist	Endodontists	Other dental specialists
Inform the patient and repair it immediately	22.2% (*n* = 6)	57.1% (*n* = 4)	37.5% (*n* = 6)
Inform the patient and repair it in another appointment	11.1% (*n* = 3)	0.0%	6.2% (*n* = 1)
Inform the patient and refer to another professional	51.9% (*n* = 14)	1% (*n* = 4)	31.2% (*n* = 5)
Do not inform the patient and continue the treatment	3.7% (*n* = 1)	1% (*n* = 4)	6.2% (*n* = 1)
Do not inform the patient and refer to another professional	11.1% (*n* = 3)	0.0%	6.2% (*n* = 1)
Other conduct	0.0%	14.3% (*n* = 1)	12.5% (*n* = 2)

## Data Availability

The data used to support the findings of this study are available from the corresponding author upon reasonable request.
